# Influence of age on the association between the triglyceride-glucose index and all-cause mortality in patients with cardiovascular diseases

**DOI:** 10.1186/s12944-022-01738-3

**Published:** 2022-12-10

**Authors:** Lu Wang, Yang Wang, Rui Liu, Lin Xu, Wen Zhong, Lijuan Li, Changyi Wang, Chengqi He, Chenying Fu, Quan Wei

**Affiliations:** 1 Rehabilitation Medicine Center and Institute of Rehabilitation Medicine, West China Hospital, Sichuan University, Chengdu, PR China; 2 Key Laboratory of Rehabilitation Medicine in Sichuan Province, Chengdu, PR China; 3 National Clinical Research Center for Geriatrics, West China Hospital, Sichuan University, Sichuan Chengdu, PR China; 4 Aging and Geriatric Mechanism Laboratory, West China Hospital, Sichuan University, Sichuan Chengdu, PR China

**Keywords:** Triglyceride-glucose index, Mortality, Age, Cardiovascular diseases, Physical activity

## Abstract

**Background:**

In patients with cardiovascular diseases, it is reported that the triglyceride-glucose index (TGI) potentially indicates prognosis. However, the results are controversial. Moreover, whether age has an impact on the predictive value of TGI remains unclear.

**Methods:**

Participants with cardiovascular diseases were enrolled using the China Health and Retirement Longitudinal Study (CHARLS) registry. TGI was calculated as ln (triglyceride×glucose/2). The survival status was recorded every 2 years in the follow-up waves. Multivariate regression analysis was carried out to determine the relationship between TGI levels and long-term all-cause mortality in patients grouped by different age. Patients younger than 65 years old were regarded as middle-aged group. Otherwise, they were classified as old group.

**Results:**

In total, 2923 patients with cardiovascular diseases and baseline blood test results were included. After 7 years of follow-up, 242 (8.91%) patients died. Cox regression analysis revealed that higher TGI levels were associated with a higher risk of long-term all-cause mortality in middle-aged participants (hazard ratio [HR], 3.64; 95% confidence interval [CI] 1.44–9.22, *P* = 0.006) but not in old participants (HR 1.20, 95% CI 0.62–2.32, *P* = 0.594, *P* for interaction = 0.017), after adjusting physical activity and other factors. Kaplan–Meier estimate analysis and restricted cubic spline curves showed similar results.

**Conclusion:**

TGI was a promising marker for predicting all-cause mortality in middle-aged patients after cardiovascular diseases. Patients younger than 65 years old who have a higher level of TGI may develop a higher risk of all-cause mortality, and they are encouraged to control vascular risk factors and take more physical activity to improve their prognosis. Additionally, whether intervention in regulating TGI levels is beneficial for the prognosis of these patients needs further investigation.

**Supplementary Information:**

The online version contains supplementary material available at 10.1186/s12944-022-01738-3.

## Background

It is well known that cardiovascular diseases are one of the most important diseases [1]. Patients with cardiovascular diseases have restricted heart function and poor quality of life, causing a serious disease burden on their families and society [2]. Exploring potentially remediable risk factors is of major significance to improve the prognosis after cardiovascular diseases [3].

Many novel risk factors, such as low insulin sensitivity, play a significant role in the prognosis of cardiovascular diseases [4–7]. Insulin resistance was related to all-cause mortality in general healthy person or patients with cardiovascular risk factors [8]. Currently, common methods used to detect insulin resistance include glucose tolerance testing and the homeostasis model assessment of insulin resistance (HOMA-IR) [9]. However, these tests are complicated and have not been commonly used in clinical practice [10–12].

Recently, the triglyceride-glucose index (TGI) has been developed as an indicator of low insulin sensitivity [13–15] and was correlated with other indicators such as HOMA-IR [16]. It was suggested that participants with higher TGI levels were predisposed to diabetes [17], hypertension [18], obesity [19], atherosclerosis [20], and cardiovascular diseases [21], all of which are significantly related to a higher risk of mortality in patients with cardiovascular diseases [22]. Recently, several studies have revealed that higher TGI levels are independently related to poorer prognosis after cardiovascular diseases [23, 24]. However, some other studies reported different results and indicated that TGI was not significantly associated with the risk of unfavorable prognosis in patients with cardiovascular diseases [25, 26]. This inconsistency may be due to the different characteristics of the participants.

TGI is associated with many factors, such as age and fasting state. It was reported that age was inversely correlated with TGI, and younger patients had higher levels of TGI [25, 27–30]. Furthermore, an increased volume of physical activity reduces the level of insulin resistance [31]. Given that younger patients typically exhibit a higher volume of physical activity than older patients [32], a complex relationship may exist between age, physical activity, and TGI. To determine whether age could modify the association between TGI and mortality and the value of physical activity in the potential interaction between age and TGI after cardiovascular diseases, a detailed analyses of data using the China Health and Retirement Registry (CHARLS) were performed.

## Methods

### Data collection

The CHARLS has been an international survey for middle-aged and old Chinese, and the data used are available by visiting the official website [33]. The initial study was launched in 2011 (wave 1), and the individuals were followed up after the initial study to explore the determinants and consequences of aging. The follow-up wave 2, wave 3, and wave 4 were released in 2013, 2015, and 2018, respectively. In total, 25,586 individuals were interviewed after combining the initial samples and refreshment samples. CHARLS collected data including past-history, health and lifestyle status, present illness, work and income, and social networks from the collected samples of 28 Chinese provinces covering 150 districts and 450 communities. The study provided detailed information for medical, social, political, and economic research. Many studies have been published based on this nationally representative database [33]. All data were obtained by interview and examination.

The inclusion criteria were 1) individuals with complete baseline data on age, gender, and history of diseases; 2) individuals with a history of cardiovascular diseases; and 3) individuals with data on plasma levels of glucose and triglycerides. Correspondingly, individuals without plasma glucose and triglycerides results were excluded. Self-reported baseline data, including demographic characteristics, chronic diseases, and lifestyle factors, were collected. Education was classified into 3 types. Specialists performed physical examination to collect body measurements [33]. Blood pressures were measured by a sphygmomanometer (Omron, Japan) three times for every participant [33]. Body mass index (BMI) was also obtained by measuring height and weight.

Cardiovascular diseases were defined as coronary artery diseases, valve heart diseases, heart failure, arrhythmia, other heart diseases, and stroke [33]. Hypertension, diabetes, dyslipidemia, and stroke were defined as past-history, or elevated blood pressure, glucose, or lipids according to diagnostic criteria, or the use of corresponding drugs [34–36]. Other chronic diseases were determined by self-reported history.

Participants responded to questions surveying the volume of physical activity. Vigorous activity includes activities such as dancing, swimming, and strenuous running. Moderate physical activity includes light housework and brisk running with harder breath than usual conditions, and mild physical activity includes walking with a normal pace and routine work with light intensity. The duration and frequency were applied to calculate the volume. For vigorous physical activity, the volume was classified as no activity, less than 75 min every week, greater than 75 and less than 300 min every week, and greater than 300 min every week [37]. Moderate and mild physical activity was classified into no activity, less than 150 min every week, greater than 150 and less than 300 min every week, and greater than 300 min every week, according to World Health Organization guideline [37].

A hematological examination was conducted by a special laboratory. Medical staff collected venous blood from participants. Blood routine tests were measured by automated analyzers within 141 min of collection. The plasma samples were put in cryovials at below 20°C, transported to Beijing, and then stored at below 80°C until assayed. Blood glucose and lipids were assayed by enzymatic colorimetric test methods [38, 39]. The detection limit of blood glucose was 2-450 mg/dl. The analytical range of triglycerides was 4-1000 mg/dl. TGI was calculated using the formula based on previous study [40]. The blood test results in wave 1 (in 2011) were regarded as baseline data. In wave 3 (in 2015), randomly collected blood sample data were regarded as follow-up data.

### Outcome assessment

The follow-up interview waves provided information on participants’ survival status (dead or alive). For participants who died before wave 2 (in 2013), the exact time and the cause of death were recorded. The survival time of these participants was evaluated by the time span between the time of recruitment in CHARLS and the exact death time. For participants who died after wave 2, only the survival status was recorded. If the time of death was unavailable, the survival time of these participants was calculated by the median of the time span between baseline time and the time of follow-up wave with death information. For participants who were alive through all waves, the survival time was the time span between the baseline wave and the last wave. Cardiovascular and non-cardiovascular mortality were defined as death due to or not due to cardiovascular diseases, respectively; non-cardiovascular mortality included death due to cancer, lung diseases, and other non-cardiovascular diseases.

### Statistical analysis

Patients were first divided into different groups with ages younger and older than 65 years (middle-aged patients and old patients). Then, the enrolled participants were further classified into the low TGI group and the high TGI group by the Receiver operating characteristic (ROC) curve. The demographic data, vascular factors, and blood test results were compared among these groups. Continuous data were displayed as the mean as well as standard deviation (SD) for normally distributed data. Variables, such as BMI, triglycerides, and C-reactive protein (CRP), were reported as the median. The difference between 2 TGI groups was conducted by a t-test or the Mann**‒**Whitney U test [21]. Categorical data are reported as numbers and proportions. Their differences were assessed by the chi-square test [21]. The effect of fasting state and cardiovascular-related drugs on the levels of triglycerides, glucose, and TGI was also explored. The spearmen correlation coefficient was also calculated for TGI and age as well as physical activity, and the distribution of physical activity were compared.

Cox regression was carried out to explore factors for all-cause mortality in middle-aged patients and old patients. Hazard ratios (HRs) as well as 95% confidence intervals (CIs) were obtained. First, univariate analysis was performed; and then multivariate analysis was performed with 2 models using potential factors according to univariate analysis (*P* < 0.05). In model 1, potential confounding factors were entered into multivariate analysis. In model 2, physical activity was further adjusted. Likelihood ratio tests were used to detect the interaction between different age groups.

To illustrate the different associations between TGI and mortality in middle-aged patients and old patients, Kaplan–Meier estimate curves and restricted cubic spline curves were generated. The differences in mortality rates were tested using the log-rank test. Then, association between fully adjusted HR for TGI levels predicting the risk of mortality in middle-aged and old groups was displayed by a restricted cubic spline curve, with four knots of TGI distribution. The median percentile of the TGI distribution was applied as a reference level [41].

To compare the prognostic value between glucose, triglycerides, and TGI, area under the curve of ROC, integrated discrimination improvement score, and net reclassification improvement score of adding glucose, triglycerides, and TGI into the original model (model 2) were calculated. To further identify whether the value of TGI on mortality in different age groups was modified by other factors, subgroup analyses including sex, diabetes, hypertension, BMI, waist circumference, physical activity, and fasting state were carried out. To explore the potential interaction between these factors and TGI levels, the *P* for interaction in each subgroup analysis was also calculated. For the sensitivity analysis, different cutoff points for age (55, 60, 65, and 70 years old) were used to recalculate HR and 95% CI. The statistical analysis was conducted by Stata 15.0 and GraphPad 8.0.

## Results

Of the 11636 participants only with plasma glucose data and 11656 participants only with plasma triglycerides data, 11636 participants had data for both glucose and triglycerides. Among them, 2923 patients with cardiovascular diseases were followed up in the subsequent waves. The average age was 60.27 ± 9.38 years, and 1151 (39.38%) patients were males. Among the 2923 patients, 2065 (70.65%) middle-aged patients were 55.59 ± 6.20 years. Furthermore, 858 (29.35%) old patients were 71.82 ± 4.72 years. The TGI in middle-aged patients (8.80 ± 0.71) was higher than that in old patients (8.73 ± 0.66, *P* = 0.007). The mean TGI was 8.78 (SD: 0.70), and the optimal cutoff value for TGI level was 8.61.

Patients without fasting had higher levels of TGI than patients with fasting (Supplementary Table 1). Patients treated with drugs for hypertension, diabetes, and dyslipidemia had higher levels of baseline TGI (in 2011) and follow-up TGI (in 2015) than those who did not (Supplementary Table 2). The TGI ranged from 4.96 to 8.61 in 1299 patients, in the low TGI group and ranged from 8.61 to 12.96 in 1624 patients, in the high TGI group. Finally, patients were divided into 4 groups.

Table 1 shows the detailed baseline data. In old patients, patients with high TGI levels (average age: 71.50) were younger than those with low TGI levels (average age: 72.15, *P* = 0.045). Additionally, in middle-aged group, the high TGI group had more male patients; in old group, the high TGI group had fewer male patients (*P* < 0.001). In both middle-aged and old groups, whose TGI levels were higher had increased levels of BMI, glucose, triglycerides, white blood cells, platelets, glycated hemoglobin, low-density lipoprotein (LDL), total cholesterol levels, and decreased levels of high-density lipoprotein (HDL). The proportions of patients with diabetes, hypertension, and dyslipidemia were also higher in the high TGI group. However, the proportions of patients with drinking and smoking habits were lower in the high TGI group. In middle-aged patients, the proportion of patients participating in vigorous physical activity was lower in the high TGI groups (*P* = 0.049).

**Table 1:** Baseline characteristics of included patients grouped by age and TGI levels

Variables	The middle-aged and low TGI group	The middle-aged and high TGI group	*P*-value	The old and low TGI group	The old and high TGI group	*P*-value
N (%)	888	1177	-	411	447	-
Age, years, mean ± SD	55.42 ± 6.33	55.71 ± 6.11	0.303	72.15 ± 4.64	71.50 ± 4.77	**0.045**
Male, n (%)	518 (58.33)	778 (66.10)	**<0.001**	225 (54.74)	157 (35.12)	**<0.001**
Education			0.439			0.614
Less than lower secondary education	781 (87.95)	1022 (86.83)		380 (92.46)	407 (91.05)	
Upper secondary education or vocational training	93 (10.47)	141 (11.98)		17 (4.14)	25 (5.59)	
Tertiary education	14 (1.58)	14 (1.19)		14 (3.41)	15 (3.36)	
SBP, mmHg, mean ± SD	129.18 ± 22.37	132.44 ± 21.48	**0.002**	135.38 ± 22.32	138.92 ± 23.12	**0.038**
BMI, kg/m^2^, median (IQR)	23.37 (21.14, 25.84)	25.51 (22.68, 28.12)	**<0.001**	21.62 (19.78, 23.92)	24.20 (21.68, 26.60)	**<0.001**
Waist circumference, cm, mean ± SD	84.36 ± 11.49	88.71 ± 14.49	**<0.001**	82.58 ± 12.71	88.64 ± 13.98	**<0.001**
Diabetes, n (%)	49 (5.57)	177 (15.25)	**<0.001**	19 (4.65)	62 (14.09)	**<0.001**
Hypertension, n (%)	297 (33.6)	561 (48.24)	**<0.001**	171 (41.81)	240 (54.3)	**<0.001**
Dyslipidemia, n (%)	106 (12.14)	307 (26.79)	**<0.001**	43 (10.59)	87 (20.00)	**<0.001**
Stroke, n (%)	23 (2.62)	47 (4.01)	0.085	21 (5.13)	22 (4.97)	0.911
Cancer, n (%)	4 (0.45)	17 (1.45)	**0.027**	6 (1.46)	4 (0.9)	0.450
Lung disease, n (%)	122 (13.9)	143 (12.22)	0.264	90 (22.06)	84 (19.05)	0.277
Liver disease, n (%)	65 (7.42)	63 (5.4)	0.062	20 (4.89)	27 (6.15)	0.423
Kidney disease, n (%)	98 (11.17)	116 (9.95)	0.370	39 (9.54)	41 (9.30)	0.905
Digestive disease, n (%)	300 (34.01)	372 (31.69)	0.266	101 (24.63)	116 (26.13)	0.617
Drinking, n (%)	329 (37.18)	349 (29.73)	**<0.001**	167 (40.73)	135 (30.54)	**0.002**
Smoking, n (%)	323 (36.5)	377 (32.09)	**0.036**	184 (44.88)	148 (33.41)	**<0.001**
Vigorous physical activity, n (%)	284 (33.33)	326 (29.19)	**0.049**	71 (19.51)	84 (22.11)	0.383
Moderate physical activity, n (%)	478 (56.10)	592 (53)	0.171	156 (42.86)	146 (38.42)	0.218
Mild physical activity, n (%)	685 (80.49)	898 (80.39)	0.956	290 (79.67)	293 (77.11)	0.396
**Laboratory tests**						
Triglycerides, mg/dl, median (IQR)	79.65 (63.72, 94.70)	161.86 (130.10, 221.25)	**<0.001**	76.11 (61.95, 91.16)	154.88 (125.67, 209.75)	**<0.001**
Glucose, mg/dl, mean ± SD	96.61 ± 16.34	125.18 ± 50.97	**<0.001**	98.09 ± 15.45	126.22 ± 49.13	**<0.001**
TGI, (mg/dl) ^2^, mean ± SD	8.20 ± 0.35	9.26 ± 0.56	**<0.001**	8.19 ± 0.30	9.22 ± 0.49	**<0.001**
TGI, (mg/dl) ^2^, range	4.96, 8.61	8.61, 12.96	**-**	6.61, 8.61	8.61, 11.66	**-**
White blood cells, 10^9^/l, mean ± SD	6.02 ± 1.81	6.39 ± 1.78	**<0.001**	5.96 ± 1.77	6.50 ± 1.97	**<0.001**
Platelets, 10^9^/l, mean ± SD	212.93 ± 69.82	223.75 ± 92.40	**0.004**	194.85 ± 86.72	207.10 ± 75.15	**0.030**
Hemoglobin, g/dl, mean ± SD	14.44 ± 2.26	14.64 ± 2.14	**0.043**	14.04 ± 2.09	14.29 ± 2.04	0.075
Hematocrit, %, mean ± SD	41.33 ± 6.32	41.76 ± 5.94	0.111	40.31 ± 6.11	41.37 ± 5.76	**0.009**
Creatinine, mg/dl, mean ± SD	0.75 ± 0.17	0.78 ± 0.21	**0.001**	0.82 ± 0.22	0.83 ± 0.24	0.456
CRP, mg/l, median (IQR)	0.89 (0.48, 1.96)	1.3 (0.72, 2.59)	0.176	1.09 (0.53, 2.38)	1.4 (0.76, 2.97)	0.156
Glycated hemoglobin, %, mean ± SD	5.12 ± 0.46	5.53 ± 1.13	**<0.001**	5.15 ± 0.48	5.52 ± 1.09	**<0.001**
HDL, mg/dl, mean ± SD	56.20 ± 14.59	43.98 ± 12.47	**<0.001**	58.26 ± 15.64	44.38 ± 13.56	**<0.001**
LDL, mg/dl, mean ± SD	114.38 ± 31.61	120.85 ± 38.64	**<0.001**	114.01 ± 33.34	124.01 ± 37.28	**<0.001**
Total cholesterol, mg/dl, mean ± SD	183.57 ± 35.22	204.31 ± 40.77	**<0.001**	185.37 ± 37.66	204.58 ± 37.84	**<0.001**
Death, n (%)	25 (3.01)	56 (5.09)	**0.024**	74 (19.73)	87 (21.17)	0.619

*Abbreviation:* *TGI* Triglyceride-glucose index, *SBP* Systaltic blood pressure, *BMI* Body mass index, *CRP* C-Reactive Protein, *HDL* High-Density Lipoprotein, *LDL* Low-Density Lipoprotein, *SD* Standard deviation, *IQR* Interquartile range

Furthermore, TGI was inversely correlated with age and moderate physical activity. Patients with old age had a lower volume of vigorous, moderate, and mild physical activity. Moreover, patients with older age and higher TGI levels had an increased rate of physical inactivity than patients with younger age and lower TGI levels (Supplementary Figs. 1-3).

After 7 years, 207 (7.08%) patients did not have information on follow-up data, and 242 (8.28%) patients died. Furthermore, in middle-aged patients, the mortality increased from 3.01% of the low TGI group to 5.09% of the high TGI group (*P* = 0.024). However, in old patients, the rate of death did not significantly differ between patients with low TGI levels and those with high TGI levels (19.73% vs. 21.17%, *P* = 0.619). Supplementary Fig. 4 shows that in middle-aged patients, cardiovascular mortality (31.87%) tended to be the dominant cause of death; while in old patients, non-cardiovascular mortality (30.49%) was more prominent.

In middle-aged patients, univariate analysis suggested that TGI was positive associated with mortality (Table 2); moreover, higher TGI levels were independently related to an increased risk of mortality (HR 2.48, 95% CI 1.28–4.85, *P* = 0.008) after adjusting for potential confounding factors. However, in old patients, TGI was not significantly related to the risk of all-cause mortality in univariate or multivariate analysis. Importantly, a significant interaction between TGI and age was seen (*P* = 0.024). Furthermore, after further adjusting for vigorous physical activity, middle-aged patients with higher TGI levels had a 2.64 times increased risk of mortality than middle-aged group with lower TGI levels (HR 3.64, 95% CI 1.44–9.22, *P* = 0.006). Nevertheless, for old patients, further adjustment only slightly changed the nonsignificant predictive value (*P* = 0.594; *P* for interaction between middle-aged and old groups: 0.017).

**Table 2:** Cox regression analysis for higher TGI levels compared with lower TGI levels predicting the risk of all-cause mortality in middle-aged and old patients

	Middle-aged patients	Old patients	*P*-value for interaction
Univariate analysis	1.70 (1.06, 2.73), **0.027**	1.09 (0.80, 1.48), 0.589	-
Multivariate analysis, Model 1	2.48 (1.28, 4.85), **0.008**	1.26 (0.78, 2.05), 0.346	**0.024**
Multivariate analysis, Model 2	3.64 (1.44, 9.22), **0.006**	1.20 (0.62, 2.32), 0.594	**0.017**

Model 1 adjusted for age, sex, systolic blood pressure, BMI, waist, diabetes, hypertension, dyslipidemia, drinking, smoking, white blood cells, platelet, creatinine, hematocrit, hemoglobin, glycated hemoglobin, HDL, LDL, total cholesterol

Model 2 adjusted for age, sex, systolic blood pressure, BMI, waist, diabetes, hypertension, dyslipidemia, drinking, smoking, white blood cells, platelet, creatinine, hematocrit, hemoglobin, glycated hemoglobin, HDL, LDL, total cholesterol, and vigorous activity

Furthermore, compare with glucose and triglycerides, TGI showed a better predictive value by using the ROC analysis and 2 discriminative scores (Supplementary Table 3). Supplementary Table 4 shows relative values of HRs were higher in the middle-aged patients and significant interactions were observed in most groups. In middle-aged groups divided by 65 and 70 years old, the association of TGI predicting all-cause mortality was significant.

**Fig. 1:**
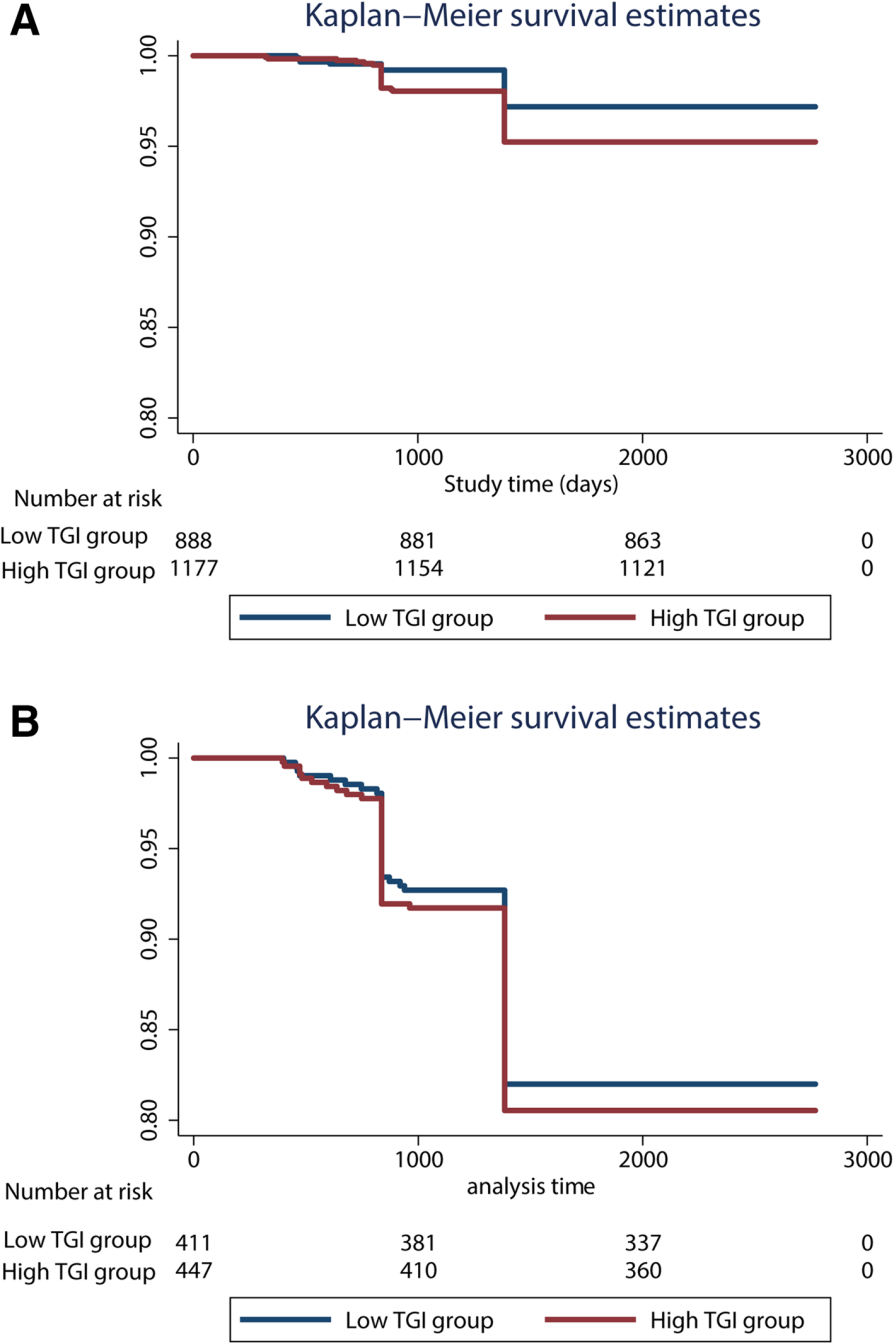
Kaplan-Meier survival estimates curve for the risk of all-cause mortality according to the higher and lower triglyceride glucose index levels in middle-aged patients (**a**) and old patients (**b**). Abbreviation: TGI, Triglyceride-glucose index

Consistently, similar results were observed in Kaplan‒Meier estimate analysis, which illustrated the estimates of mortality among middle-aged patients (Fig. 1a) and old patients (Fig. 1b). In middle-aged patients, compared with the low TGI group, the risk of mortality was significantly higher in the high TGI group (Fig. 1a, *P* for log-rank test = 0.024). However, in old patients, the *P* for log-rank test was 0.573, suggesting insignificant results (Fig. 1b).

**Fig. 2:**
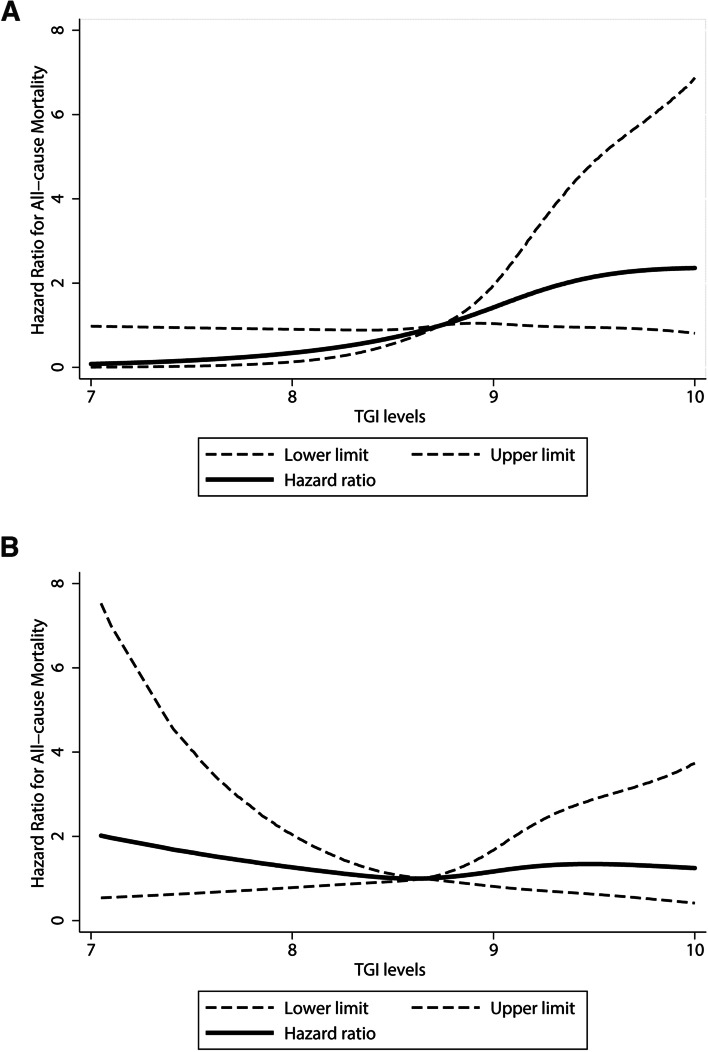
Adjusted dose-response association between triglyceride glucose index levels and the risk of all-cause mortality in middle-aged patients (**a**) and old patients (**b**). TGI was coded using a restricted cubic spline function with four knots located at the 25th, 50th, 75th, and 95th percentiles of the distribution of TGI. Reference TGI value is 8.74 in (**a**) and 8.65 in (**b**). Y-axis represents the adjusted hazard ratios of all-cause mortality risk when comparing patients with any value of TGI with patients with the reference value of TGI. Dashed lines are 95% confidence intervals. Adjusted factors included age, sex, systolic blood pressure, BMI, waist, diabetes, hypertension, dyslipidemia, drinking, smoking, white blood cells, platelet, creatinine, hematocrit, hemoglobin, glycated hemoglobin, HDL, LDL, total cholesterol, and vigorous physical activity. Abbreviation: TGI, triglyceride glucose index, BMI, body mass index, HDL, high-density lipoprotein, LDL, low-density lipoprotein

To identify the linear relationship in middle-aged and old patients, a spline curve was generated to delineate the change in mortality. Figure 2a shows that in middle-aged patients, HRs increased along with TGI levels, suggesting an elevated risk of mortality with increases in the magnitude of TGI levels. However, in old patients, no dose-response relationship was observed (Fig. 2b).

To explore whether sex, diabetes, hypertension, BMI, waist circumference, and vigorous physical activity interact with TGI to predict all-cause mortality, stratified analyses were performed using these factors, and the stratified factor was not adjusted in each analysis. No additional variables that modified the association between TGI levels and mortality in middle-aged and old patients were identified (Table 3). In middle-aged patients, TGI was a predictor of mortality in male patients, patients without diabetes, patients with larger BMI, patients with larger waist circumference, patients without vigorous physical activity, and patients with fasting TGI. Nevertheless, in old patients, TGI was significantly related to mortality only in patients with BMI greater than 24 kg/m^2^.

**Table 3:** Stratified Cox regression analysis to identify variables that modify the correlation between triglyceride glucose index values and the risk of all-cause mortality

Subgroups	Middle-aged patients		Old patients	
	Hazard ratio (95% confidence interval)	*P*-value for interaction	Hazard ratio (95% confidence interval)	*P*-value for interaction
Sex		0.100		0.325
Male	4.21 (1.56, 11.39),**0.005**		0.96 (0.48, 1.93), 0.914	
Female	1.82 (0.72, 4.58), 0.205		2.11 (0.95, 4.68), 0.067	
Diabetes		0.459		0.060
Yes	3.27 (0.41, 25.73),0.261		1.68 (0.13, 20.98),0.689	
No	2.34 (1.17, 4.69),**0.016**		1.20 (0.71, 2.03),0.496	
Hypertension		0.852		0.457
Yes	2.79 (0.98, 7.94), 0.055		1.45 (0.70, 3.03), 0.318	
No	2.41 (0.97, 5.98), 0.058		1.06 (0.54, 2.10), 0.860	
BMI, kg/m^2^		0.887		0.722
<24	1.93 (0.70, 5.34), 0.205		1.13 (0.63, 2.00), 0.684	
≥24	3.14 (1.21, 8.20), **0.019**		3.21 (1.04, 9.88), **0.043**	
Waist, cm		0.826		0.992
<85	2.46 (0.82,7.43), 0.109		1.04 (0.51, 2.12), 0.913	
≥85	2.91 (1.21, 6.99), **0.017**		1.46 (0.70, 3.02), 0.312	
Vigorous physical activity		0.798		0.596
Yes	1.39 (0.23, 8.35),0.720		0.62 (1.13, 2.87),0.543	
No	3.45 (1.27, 9.31),**0.015**		1.27 (0.63, 2.53),0.504	
Fasting		0.783		0.285
Yes	4.29 (1.46, 12.64), **0.008**		1.11 (0.56, 2.20), 0.764	
No	1.31 (0.25, 6.82), 0.750		3.01 (0.37, 24.50), 0.302	

*Abbreviation:* *TGI* Triglyceride glucose index, *BMI* Body mass index

## Discussion

This study suggested higher TGI levels were significantly related to a higher risk of all-cause mortality in middle-aged patients. The association was only significant in middle-aged patients, but not in old patients. A significant interaction was observed between TGI and age for predicting the risk of all-cause mortality.

Additionally, considering that physical activity was one of the main influencing factors of insulin resistance, the relationship among age, physical activity, and TGI was also explored. TGI levels were associated with poor prognosis regardless of whether physical activity was adjusted. In the subgroup analysis, the association was significant in middle-aged patients without vigorous physical activity. These results suggested that patients without adequate physical activity may have a severe degree of insulin resistance, which may increase the risk of mortality. Therefore, middle-aged patients should perform more physical activity to potentially reduce the risk of poor prognosis after cardiovascular diseases. Further studies should explore whether participating in more physical activity could decrease TGI levels and thus improve prognosis in middle-aged patients with cardiovascular diseases.

Consistent with the present study, Ma et al. found participates with elevated TGI levels had a higher rate of major adverse cardiovascular events in middle-aged patients but not in old patients [27]. However, subgroup analysis for all-cause mortality stratified by age was not performed [27]. Interestingly, although Yang et al. revealed that TGI levels may be associated with unfavorable prognosis, they found different results in patients of different ages grouped with 65 years old [25]. TGI tended to be a marker of good prognosis in younger patients, and an indicator of poor prognosis in older patients [25].

In contrast, the present study indicated that TGI was a predictor of prognosis exclusively in middle-aged patients, with a significant interaction between age and TGI. This differs from those of Yang et al. given the following possible explanation. First, the patients included were different. All kinds of cardiovascular diseases were included in this study. Nevertheless, Yang et al. only selected patients without diabetes who received PCI. Patients who undergo acute surgery are susceptible to hyperglycemia, which may have an impact on the true effect of TGI on outcomes [42]. Second, the outcome measurements were different. Yang et al. included 2-year major adverse cardiovascular events as outcome indicators. The rate of all-cause mortality in their study was relatively low (1%). Thus, it was impossible to directly compare the present study with their studies. Third, age has an impact on the degree of insulin resistance, which may partially explain the complex role of age in the association between TGI and prognosis. It has been reported that younger patients are more susceptible to insulin resistance [25], and have higher TGI levels [25, 27–30]. Younger participants with high levels of TGI may have an elevated risk of insulin resistance-related diseases such as diabetes and obesity, which may increase the risk of mortality [43]. Thus, TGI was a significant marker to represent the risk of mortality in relatively young participants.

Of note, it was found no significant results for TGI predicting mortality in old patients, and the reason was unclear. In this study, old patients had more proportion of non-cardiovascular mortality. Previously, TGI was a marker of cardiovascular diseases and cardiovascular mortality [44]. Therefore, the weak relationship between TGI and non-cardiovascular mortality may partly explain the unrelatedness in old patients. It is worth investigating whether the lower distinguishing ability of TGI in older patients is due to the different predictive abilities of TGI for cardiovascular and non-cardiovascular mortality.

Previously, studies have suggested that higher TGI levels were related to an elevated risk of poor outcomes in participants with coronary artery diseases [45, 46], patients with nonobstructive coronary arteries [47], patients with coronary heart diseases [27, 44]. These studies suggested the potential role of TGI levels in predicting clinical outcomes after cardiovascular diseases.

However, other studies proposed that TGI levels were not independently related to outcomes [25, 26, 48]. Vega et al. suggested that TGI levels were not related to mortality in the general male population [48]. Apparently, the results exhibited gender selection bias. Another study included 1340 myocardial infarction patients without diabetes, and concluded that TGI was not an indicator of mortality [26]. Furthermore, Wang et al. suggested that TGI was not correlated with mortality but was associated with major adverse cardiovascular events [44]. The low rate of mortality in their study made it difficult to identify the association. Additionally, Zhao et al. indicated that Kaplan–Meier curves of poor prognosis did not significantly differ between groups with different TGI levels [12]. Numerous differences in patient selection, outcome definition, and study design were noted in previous studies, all of which may contribute to the inconsistency of these results. Therefore, this calls for more studies to explore the potential modifiers.

Several possible mechanisms may explain the positive association [9]. TGI reflects hyperglycemia and hyperlipidemia. Higher blood glucose could induce oxidative stress, causing endothelial dysfunction [49–51]. Elevated plasma lipids promote the formation of atherosclerotic plaques [52]. In addition, hyperglycemia and dyslipidemia increase thrombotic events to increase the risk of atherosclerotic plaque rupture [20], cardiovascular event recurrence [53–55] and mortality [53, 56]. The aforementioned mechanisms might partly give the reason for the potential relationship between TGI and mortality risk in patients with cardiovascular diseases.

Previously, many research have revealed the association between low insulin sensitivity and poor outcome [57, 58]. Insulin resistance is related to energy dysmetabolism, oxidative stress, vascular remodeling, and poor endothelial function, all of which result in higher myocardial oxygen consumption, lower myocardial compensatory capacity, and reduced collateral formation [59–64]. These factors lead to poorer myocardial reperfusion and coronary microcirculation, larger infarct size, and more adverse cardiovascular events [65].

Moreover, traditional cardiovascular risk factors may be the bridge between TGI and mortality. TGI is also associated with BMI, waist circumference, glycated hemoglobin, LDL, and total cholesterol levels, indicating that patients with higher TGI have more cardiovascular risk factors, which may lead to poor prognosis of patients with cardiovascular diseases. Finally, TGI may increase the severity of cardiovascular diseases to increase the risk of death. Higher TGI levels are associated with more serious cardiovascular diseases, coronary calcification and stenosis, atheroma plaque formation rupture, micro- and macroangiopathies, and cardiac autonomic neuropathy [66–68], and all of these conditions increase the incidence of adverse cardiovascular events. Participants with higher TGI levels had an increased risk of acute vascular events [21], thus increasing the risk of mortality.

Of note, TGI is a simple, low-cost, and routinely measured surrogate indicator that reflects the long-term risk of mortality compared with other markers. Thus, TGI may exhibit promise promising to be applied in future clinical applications to help the early selection of middle-aged patients who have a high risk of mortality. In short, TGI may provide critical value for risk stratification in middle-aged patients. Patients with a higher TGI level should be closely monitored to evaluate the risk of mortality. Another important consideration for the application of TGI is the influence of other factors, such as the fasting state and weight. Fasting TGI may have a better predictive value than non-fasting TGI. Although the interaction between TGI and fasting state as well as BMI in the present study was not significant, many studies have explored the predictive value of combining TGI with other markers such as TGI combining with BMI [69]. Therefore, other factors related to TGI should also be considered before the usage of TGI in clincial practice.

Importantly, decreasing the levels of insulin resistance may benefit clinical prognosis of patients with cardiovascular disease [70]. Although previous studies have suggested many strategies to reduce the level of TGI, the role of these strategies in improving prognosis remains unknown. For example, a special diet, such as whole-grain consumption, decreased the level of insulin resistance [71], and high carbohydrate consumption and low lipid consumption reduced the risk of developing high TGI levels [72]. It is unclear whether intervention to lower TGI could improve the prognosis of these patients and whether TGI can serve as an indicator to evaluate the role of therapies on insulin resistance intervention. Additionally, it is unclear the effect of cardiovascular diseases related drugs on the levels of TGI. This post-hoc analysis suggested patients using drugs for hypertension, diabetes, and dyslipidemia had higher TGI levels. However, the inverse cause-effect relationship may exist. Whether therapies, such as statins and antidiabetic drugs, could decrease the levels of TGI to improve the prognosis in patients with high TGI levels remains to be investigated by high-quality clinical randomized control trials [73].

Furthermore, it is worth noting that other biomarkers such as hemoglobin [74] and troponin [75] also show great predictive value for patients with cardiovascular diseases. Previously, researchers have established some predictive models with clinical factors and biomarkers to evaluate the prognosis [76, 77], there need more studies to investigate whether combining TGI with other promising biomarkers could improve the predictive value of these prediticve models.

### Strengths and limitations

The present study provides novel information for the potential application of TGI in middle-aged patients. Moreover, It is insipiring to propose that age may be a potential modifier for TGI predicting mortality after cardiovascular diseases.

However, it also has several limitations. First, middle-aged and old adults were included. Therefore, it is unclear whether the significant association between TGI and age also occurred in individuals younger than 45 years old. The mortality in young patients was low, further investigations are needed to figure out the value of TGI in a relatively low-mortality rate population. Additionally, other insulin-resistance markers, such as HOMA-IR, were not compared with TGI in the prediction of mortality. However, considering that biochemical tests are more widely used, TGI may be a more easily obtained and promising routinely used marker for predicting mortality than other markers.

Moreover, the data on the cause of death was unavailable in some patients, so the association between TGI and different cause of mortality were not analyzed. TGI is a marker of cardiovascular diseases and cardiovascular mortality [78], and whether TGI is also related to non-cardiovascular mortality is unclear. Therefore, it is worth investigating the ability of TGI to distinguish different causes of mortality. Lastly, the study included participants with all types of cardiovascular diseases. The datasets had no information on some certain types of cardiovascular diseases influenced by insulin resistance such as arteriosclerotic cardiovascular disease (ASCVD), further studies should be conducted to clarify whether TGI had a better prognostic value for ASCVD than other types of cardiovascular diseases.

## Conclusion

TGI was a promising marker for predicting all-cause mortality in middle-aged patients after cardiovascular diseases. Patients younger than 65 years old who have a higher level of TGI may develop a higher risk of all-cause mortality, and they are encouraged to control vascular risk factors and take more physical activity to improve their prognosis. Additionally, whether intervention in regulating TGI levels is beneficial for the prognosis of these patients needs further investigation.

## Supplementary Information

**Additional file 1: Supplementary Table 1.** The value of triglycerides, glucose, and TGI grouped by fasting state in middle-aged and old patients. **Supplementary Table 2.** The effect of cardiovascular-related drugs on the levels of triglycerides, glucose, and TGI. **Supplementary Table 3.** The difference of predictive values for triglycerides, glucose, TGI in middled-aged and old patients. **Supplementary Table 4.** The association between TGI and all-cause mortality grouped by different cutoff values for ages. **Supplementary Table 5.** Cox regression analysis for TGI levels as continuous variables predicting the risk of all-cause mortality in middle-aged and old patients. **Supplementary Figure 1.** The proportion of vigorous physical activity volumes in patients with different age and TGI levels. **Supplementary Figure 2.** The proportion of moderate physical activity volumes in patients with different age and TGI levels. **Supplementary Figure 3.** The proportion of mild physical activity volumes in patients with different age and TGI levels. **Supplementary Figure 4.** The proportion of cardiovascular mortality and non-cardiovascular mortality in middle-aged and old patients. 

## Data Availability

The data are available from http://charls.pku.edu.cn.

## Abbreviations

TGI: Triglyceride-glucose index
HOMA-Insulin Resistance: The homeostasis model assessment of IR
CHARLS: The China Health and Retirement Registry
BMI: Body Mass Index
SBP: Systolic blood pressure
DBP: Diastolic blood pressure
HDL: High-density lipoprotein
LDL: Low-density lipoprotein
ROC: Receiver Operating Characteristic curve
SD: Standard deviation
CRP: C-Reactive Protein
HRs: Hazard ratios
CIs: Confidence intervals
PCI: Percutaneous coronary intervention
ASCVD: Arteriosclerotic cardiovascular disease
