# Supplementary material for: Influence of age on the association between the triglyceride-glucose index and all-cause mortality in patients with cardiovascular diseases
Source: Lipids Health Dis. 2022 Dec 10;21:135. doi: 10.1186/s12944-022-01738-3 (PMC9741797; doi:10.1186/s12944-022-01738-3)
Supplement: Supplementary file 1 — Additional file 1: Supplementary Table 1. The value of triglycerides, glucose, and TGI grouped by fasting state in middle-aged and old patients. Supplementary Table 2. The effect of cardiovascular-related drugs on the levels of triglycerides, glucose, and TGI. Supplementary Table 3. The difference of predictive values for triglycerides, glucose, TGI in middled-aged and old patients. Supplementary Table 4. The association between TGI and all-cause mortality grouped by different cutoff values for ages. Supplementary Table 5. Cox regression analysis for TGI levels as continuous variables predicting the risk of all-cause mortality in middle-aged and old patients. Supplementary Figure 1. The proportion of vigorous physical activity volumes in patients with different age and TGI levels. Supplementary Figure 2. The proportion of moderate physical activity volumes in patients with different age and TGI levels. Supplementary Figure 3. The proportion of mild physical activity volumes in patients with different age and TGI levels. Supplementary Figure 4. The proportion of cardiovascular mortality and non-cardiovascular mortality in middle-aged and old patients. [file 12944_2022_1738_MOESM1_ESM.docx]

**Supplementary Table 1. The value of** **triglycerides, glucose, and TGI grouped by fasting state in middle-aged and old patients.**

|  | **Middle-aged patients** | | | |  | | | | **Old patients** | | | |  |
| --- | --- | --- | --- | --- | --- | --- | --- | --- | --- | --- | --- | --- | --- |
|  | **Numbers** | | **values** | | |  | | | **Numbers** | | **values** | |  |
| Triglycerides, mg/dl, median (IQR) |  |  | |  | | | |  | |  | |  |  |
| Fasting | 1850 | 115.94 (82.31, 170.81) | |  | | | | 784 | | 106.64 (76.11, 153.99) | |  |  |
| Non-fasting | 168 | 138.06 (92.04, 209.75) | |  | | | | 52 | | 126.56 (88.5, 194.26) | |  |  |
| ***P*-value** |  | **0.001** | |  | | | |  | | **0.028** | |  |  |
| Glucose, mg/dl, mean ± SD |  |  | |  | | | |  | |  | |  |  |
| Fasting | 1850 | 112.43 ± 38.06 | |  | | | 784 | | | 112.06 ± 37.45 | |  |  |
| Non-fasting | 168 | 124.79 ± 74.85 | |  | | | 52 | | | 122.37 ± 46.08 | |  | |
| ***P*-value** |  | **＜0.001** | |  | | |  | | | 0.059 | |  |  |
| TGI, (mg/dl) ^2^, mean ± SD |  |  | |  | | | |  | |  | |  |  |
| Fasting | 1850 | 8.80 ± 0.69 | |  | | | | 784 | | 8.71 ± 0.64 | |  |  |
| Non-fasting | 168 | 8.95 ± 0.87 | |  | | | | 52 | | 8.94 ± 0.68 | |  |  |
| ***P*-value** |  | **0.006** | |  | | | |  | | **0.015** | |  |  |

Abbreviation: SD, standard deviation; TGI, triglyceride glucose index.

**Supplementary Table 2. The effect of cardiovascular-related drugs on the levels of** **triglycerides, glucose, and TGI.**

| **Types of Drugs** | **With** | **Without** | ***P*-value** |
| --- | --- | --- | --- |
| **Anti-hypertension** |  |  |  |
| Glucose, mg/dl, mean ± SD | 116.71 ± 43.95 | 110.94 ± 40.44 | **＜0.001** |
| Triglycerides, mg/dl, median (IQR) | 130.10 (88.5, 186.74) | 107.97 (78.77, 160.19) | **＜0.001** |
| TGI, (mg/dl) ^2^, mean ± SD | 8.91 ± 0.72 | 8.71 ± 0.68 | **＜0.001** |
| Follow-up Glucose, mg/dl, mean ± SD | 111.08 ± 44.06 | 101.77 ± 36.04 | **＜0.001** |
| Follow-up Triglycerides, mg/dl, median (IQR) | 136.28 (95.58, 197.35) | 115.93 (84.96, 166.37) | **＜0.001** |
| Follow-up TGI, (mg/dl) ^2^, mean ± SD | 8.92 ± 0.67 | 8.70 ± 0.63 | **＜0.001** |
| **Anti-diabetes** |  |  |  |
| Glucose, mg/dl, mean ± SD | 169.96 ± 61.33 | 112.03± 40.77 | **＜0.001** |
| Triglycerides, mg/dl, median (IQR) | 125.67 (90.27, 200.90) | 114.17 (81.42, 169.04) | 0.260 |
| TGI, (mg/dl) ^2^, mean ± SD | 9.29 ± 0.72 | 8.77 ± 0.70 | **＜0.001** |
| Follow-up Glucose, mg/dl, mean ± SD | 185.74 ± 82.14 | 103.95 ± 36.41 | **＜0.001** |
| Follow-up Triglycerides, mg/dl, median (IQR) | 138.94 (89.38, 273.45) | 122.12 (89.38, 177.88) | 0.122 |
| Follow-up TGI, (mg/dl) ^2^, mean ± SD | 9.45 ± 0.90 | 8.78 ± 0.64 | **＜0.001** |
| **Anti-dyslipidemia** |  |  |  |
| Glucose, mg/dl, mean ± SD | 125.09 ± 49.53 | 111.56 ± 40.75 | **＜0.001** |
| Triglycerides, mg/dl, median (IQR) | 142.49 (101.78, 223.91) | 110.63 (80.54, 163.73) | **＜0.001** |
| TGI, (mg/dl) ^2^, mean ± SD | 9.11 ± 0.75 | 8.74 ± 0.68 | **＜0.001** |
| Follow-up Glucose, mg/dl, mean ± SD | 117.69 ± 49.87 | 103.34 ± 37.13 | **＜0.001** |
| Follow-up Triglycerides, mg/dl, median (IQR) | 148.67 (103.54, 221.24) | 118.58 (87.61, 170.80) | **＜0.001** |
| Follow-up TGI, (mg/dl) ^2^, mean ± SD | 9.07 ± 0.73 | 8.74 ± 0.63 | **＜0.001** |
| **For cardiovascular diseases** |  |  |  |
| Glucose, mg/dl, mean ± SD | 113.20 ± 39.27 | 112.77 ± 42.68 | 0.803 |
| Triglycerides, mg/dl, median (IQR) | 119.48 (83.19, 179.66) | 112.40 (80.54, 168.15) | 0.050 |
| TGI, (mg/dl) ^2^, mean ± SD | 8.82 ± 0.72 | 8.77 ± 0.69 | 0.070 |
| Follow-up Glucose, mg/dl, mean ± SD | 106.28 ± 41.38 | 105.56 ± 39.08 | 0.693 |
| Follow-up Triglycerides, mg/dl, median (IQR) | 127.43 (89.38, 186.73) | 120.35 (88.50, 172.57) | 0.117 |
| Follow-up TGI, (mg/dl) ^2^, mean ± SD | 8.82 ± 0.68 | 8.78 ± 0.65 | 0.190 |
| **For stroke** |  |  |  |
| Glucose, mg/dl, mean ± SD | 112.89 ± 27.49 | 112.90 ± 41.93 | 0.998 |
| Triglycerides, mg/dl, median (IQR) | 129.21 (96.02, 212.84) | 114.17 (81.42, 169.04) | 0.160 |
| TGI, (mg/dl) ^2^, mean ± SD | 8.90 ± 0.71 | 8.78 ± 0.70 | 0.199 |
| Follow-up Glucose, mg/dl, mean ± SD | 114.35 ± 40.18 | 105.58± 39.99 | 0.082 |
| Follow-up Triglycerides, mg/dl, median (IQR) | 121.24 (91.15, 199.12) | 123.01 (89.38, 178.76) | 0.533 |
| Follow-up TGI, (mg/dl) ^2^, mean ± SD | 8.93 ± 0.72 | 8.79 ± 0.66 | 0.107 |

Abbreviation: SD, standard deviation; IQR, interquartile range.

**Supplementary Table 3. The difference of predictive values for triglycerides, glucose, TGI in middled-aged and old patients.**

| **Variables** | **Middle-aged patients** | **Old patients** |
| --- | --- | --- |
| **AUC of ROC (95% CI)** |  |  |
| Triglycerides | 0.550 (0.488, 0.612) | 0.488 (0.436, 0.541) |
| Glucose | 0.581 (0.519, 0.644) | 0.508 (0.453, 0.561) |
| TGI | 0.601 (0.536, 0.666) | 0.537 (0.483, 0.591) |
| *P*-value | **0.017** | 0.138 |
| **Scores for integrated discrimination improvement, *P*-value** |  |  |
| original model + triglycerides | 0.0002 ± 0.0003, 0.493 | 0.0008 ± 0.001, 0.491 |
| original model + glucose | 0.003 ± 0.002, 0.087 | 0.002 ± 0.002, 0.329 |
| original model + TGI | 0.003 ± 0.001, **0.041** | 0.016 ± 0.006, **0.010** |
| **Scores for net reclassification improvement, *P*-value** |  |  |
| original model + triglycerides | 0.197 ± 0.114, 0.083 | 0.063 ± 0.089, 0.481 |
| original model + glucose | 0.223 ± 0.114, 0.050 | 0.121 ± 0.089, 0.180 |
| original model + TGI | 0.251 ± 0.114, **0.027** | 0.278 ± 0.089, **0.002** |

Abbreviation: AUC, area under curve; ROC, receiver operating characteristic; TGI, triglyceride glucose index; CI: confidence interval. Original model includes variables adjusted in model 2 of multivariate analysis.

**Supplementary Table 4. The association between TGI and all-cause mortality grouped by different cutoff values for ages.**

|  | Middle-aged patients | |  | |  |
| --- | --- | --- | --- | --- | --- |
|  | Numbers | Hazard ratio (95% CI) | *P*-value for interaction | |  |
| **Grouped by 55 years old** |  |  | **0.018** | |  |
| Middle-aged patients | 825 | 6.10 (0.63, 58.56), 0.117 |  |  |  |
| Old patients | 2098 | 1.60 (0.95, 2.71), 0.079 |  |  | |
| **Grouped by 60 years old** |  |  | 0.243 | |  |
| Middle-aged patients | 1412 | 3.07 (0.82, 11.51), 0.096 |  | |  |
| Old patients | 1511 | 1.59 (0.92, 2.76), 0.098 |  | |  |
| **Grouped by 65 years old** |  |  | **0.017** | |  |
| Middle-aged patients | 2065 | 3.64 (1.44, 9.22), **0.006** |  | |  |
| Old patients | 858 | 1.20 (0.62, 2.32), 0.594 |  | |  |
| **Grouped by 70 years old** |  |  | **0.028** | |  |
| Middle-aged patients | 2389 | 3.92 (1.73, 8.88), **＜0.001** |  | |  |
| Old patients | 534 | 0.90 (0.42, 1.92), 0.785 |  | |  |

Adjusted for age, sex, systolic blood pressure, BMI, waist, diabetes, hypertension, dyslipidemia, drinking, smoking, white blood cells, platelet, creatinine, hematocrit, hemoglobin, glycated hemoglobin, HDL, LDL, total cholesterol, and vigorous activity.

**Supplementary Table 5. Cox regression analysis for TGI levels as continuous variables predicting the risk of all-cause mortality in middle-aged and old patients.**

|  | Middle-aged patients | Old patients | *P*-value for interaction |
| --- | --- | --- | --- |
| Univariate analysis | 1.35 (1.02, 1.79), **0.034** | 1.12 (0.89, 1.42), 0.335 | - |
| Multivariate analysis, Model 1 | 2.68 (1.52, 4.72), **0.001** | 1.04 (0.65, 1.65), 0.879 | **0.039** |
| Multivariate analysis, Model 2 | 2.57 (1.39, 4.77), **0.003** | 0.93 (0.54, 1.59), 0.787 | **0.042** |

Model 1 adjusted for age, sex, systolic blood pressure, BMI, waist, diabetes, hypertension, dyslipidemia, drinking, smoking, white blood cells, platelet, creatinine, hematocrit, hemoglobin, glycated hemoglobin, HDL, LDL, total cholesterol.

Model 2 adjusted for age, sex, systolic blood pressure, BMI, waist, diabetes, hypertension, dyslipidemia, drinking, smoking, white blood cells, platelet, creatinine, hematocrit, hemoglobin, glycated hemoglobin, HDL, LDL, total cholesterol, and vigorous activity.

**Supplementary Figure 1 The proportion of vigorous physical activity volumes in patients with different age and TGI levels.**

Abbreviation: min/w: minutes per week.

**Supplementary Figure 2 The proportion of moderate physical activity volumes in patients with different age and TGI levels.**

Abbreviation: min/w: minutes per week.

**Supplementary Figure 3 The proportion of mild physical activity volumes in patients with different age and TGI levels.**

Abbreviation: min/w: minutes per week.

**Supplementary Figure 4 The proportion of cardiovascular mortality and non-cardiovascular mortality in middle-aged and old patients.**
